# A Novel Clustering Methodology Based on Modularity Optimisation for Detecting Authorship Affinities in Shakespearean Era Plays

**DOI:** 10.1371/journal.pone.0157988

**Published:** 2016-08-29

**Authors:** Leila M. Naeni, Hugh Craig, Regina Berretta, Pablo Moscato

**Affiliations:** 1 The Priority Research Centre of Bioinformatics and Information-Based Medicine, The University of Newcastle, Newcastle, New South Wales, Australia; 2 School of Electrical Engineering and Computer Science, Faculty of Engineering and Built Environment, The University of Newcastle, Newcastle, New South Wales, Australia; 3 School of Built Environment, Faculty of Design, Architecture and Building, University of Technology Sydney, Sydney, Australia; 4 Centre for Literary and Linguistic Computing, School of Humanities and Social Science, The University of Newcastle, Newcastle, New South Wales, Australia; New York University, UNITED STATES

## Abstract

In this study we propose a novel, unsupervised clustering methodology for analyzing large datasets. This new, efficient methodology converts the general clustering problem into the community detection problem in graph by using the Jensen-Shannon distance, a dissimilarity measure originating in Information Theory. Moreover, we use graph theoretic concepts for the generation and analysis of proximity graphs. Our methodology is based on a newly proposed memetic algorithm (iMA-Net) for discovering clusters of data elements by maximizing the modularity function in proximity graphs of literary works. To test the effectiveness of this general methodology, we apply it to a text corpus dataset, which contains frequencies of approximately 55,114 unique words across all 168 written in the Shakespearean era (16^th^ and 17^th^ centuries), to analyze and detect clusters of similar plays. Experimental results and comparison with state-of-the-art clustering methods demonstrate the remarkable performance of our new method for identifying high quality clusters which reflect the commonalities in the literary style of the plays.

## Introduction

Following great advancements in technology, all scientific fields have been faced with huge empirical datasets. Identifying groups of objects, patterns or elements with *“similar characteristics”* has always drawn the attractions of researchers. *Data clustering* is one of the most widely studied problems in data mining and machine learning with a wide variety of applications in many fields ranging from the analysis of genomic data in biology [1, 2] to classifying customers for efficient marketing strategies [3, 4]. Clusters are *natural* groups in data such that elements within the same cluster are more similar to each other than to elements belonging to other clusters [5]. Thousands of techniques have been proposed to address a wide variety of clustering problems in different disciplines (see the review of data mining and clustering techniques by Han and Kamber [6]). The main clustering methods include probabilistic and generative models [7, 8], distance-based clustering [9], density and grid-based clustering [10, 11], spectral clustering [12, 13] and graph clustering [14].

*Graph clustering* is the process of grouping vertices of a graph into clusters according to the structure of the graph. Generally speaking, a good graph clustering contains clusters with many edges within the clusters and few between clusters. However this definition is unclear, thus many quality functions have been proposed to evaluate graph clusters such as *ratio cut* [15], *normalized cut* [16] and *modularity* [17]. In the recent literature a cluster of vertices in a graph is mostly called a *community* [18] and graph clustering discussed as the *community detection problem* [19]. The graph clustering problem is a fundamental algorithmic problem, however it has not been solved satisfactory and it is a notably challenging member of the NP-complete class [20, 21].

This study proposes a new data clustering methodology for the authorship analysis in a dataset generated from 168 plays from the Shakespearean era. Authorship study is a long-standing research activity, predating the availability of computers by many centuries. Interest in authorship follows from the magnitude of the consequences of assigning a work to one author or another, e.g. in separating canonical and apocryphal books in a religious tradition, establishing the authenticity of legal documents, or determining which works or parts of works are by celebrated literary authors. With the availability of digital text and computational tools, this activity is now predominantly quantitative. The Shakespearean canon has been a particular focus, with studies testing the claims of various possible authors other than William Shakespeare of Stratford to the works we know as Shakespeare’s [22], and debating possible additions to Shakespeare’s canon [23, 24]. The advent of quantitative methods has also renewed interest in the idea that authorship is objectively a property of texts, rather than largely the invention of readers and institutions, as was argued by poststructuralist theorists [25–28].

Here, we propose a new methodology for identifying groups in the large text corpus dataset. The methodology is easily generalizable and can be employed for analyzing any other dataset. This advanced clustering methodology brings together *Jensen-Shannon divergence*(JSD) as an information theoretic dissimilarity measure and combines it with a newly proposed memetic algorithm (iMA-Net) for graph clustering to yield a powerful strategy for segmenting the text corpus dataset. The proposed memetic algorithm maximizes the modularity quality function and it is an unsupervised algorithm, i.e. the method does not require any information about the data elements, such as authorship and genre of plays, it only uses the structure of the dataset to uncover clusters of similar plays. The outcomes are promising and show great authorial affinities in each cluster; a computational proof of the highly individual literary style of each author.

## Materials and Methods

### Dataset

In this work, we utilized a text corpus dataset containing 168 plays from the Shakespearean era (16^th^ and 17^th^ centuries) with the unambiguous contribution of authorship of 39 authors (see Table 1). This dataset was generated by Craig and Whipp [29] using a software application called *Intelligent Archive* (IA) to count variant spellings of approximately 55,114 unique words across all 168 plays. The software tool IA is used to extract the word-play matrix from a collection of suitably edited texts, so that only the words of dialog are counted and other materials, such as prefaces, stage directions and speaker tags are omitted. In the texts, contractions such as “they’re” are expanded so this counts as one instance of *they* and one of *are*. The IA has the capacity to combine variant spelling forms so that multiple spellings of the same word are combined into a single count [29]. As a result, this dataset contains about 9.3 million word usage statistics (word frequencies) stored in the form of a (55,114 × 168) matrix. The word-play matrix is available in the supporting information of [30].

**Table 1:** Authors and their contributions in the 168 Shakespearean era plays dataset.

Author	# Plays	Author	# Plays	Author	# Plays
Shakespeare	28	Shirley	3	Greville	1
Middleton	18	Webster	3	Davenant	1
Jonson	17	Rowley	2	Brome	1
Fletcher	15	Massinger	2	Day	1
Chapman	13	Haughton	2	Porter	1
Lyly	8	Kyd	2	Chettle	1
Ford	7	Marston	2	Edwards	1
Peele	5	Wilmot	1	Suckling	1
Dekker	5	Carey	1	Sidney	1
Marlowe	5	Daniel	1	Marmion	1
Heywood	5	Brandon	1	Beaumont	1
Greene	4	Lodge	1	Tourneur	1
Wilson	3	Goffe	1	Nashe	1

This dataset was analyzed by Marsden et al. [30] and they found measurably distinct literary styles by detecting the tendency towards overuse or avoidance of particular words for four key authors who account for the largest number of plays in the dataset. That study resulted in a set of marker words that can distinguish between plays written by Fletcher, Jonson, Middleton and Shakespeare compared to the remainder of the authors. Recently, Arefin et al. [31] used this dataset to test the performance of their new graph clustering method incorporating paraclique identification and a clustering method known as MST-*k*NN [32].

### Method

**Fig 1:**
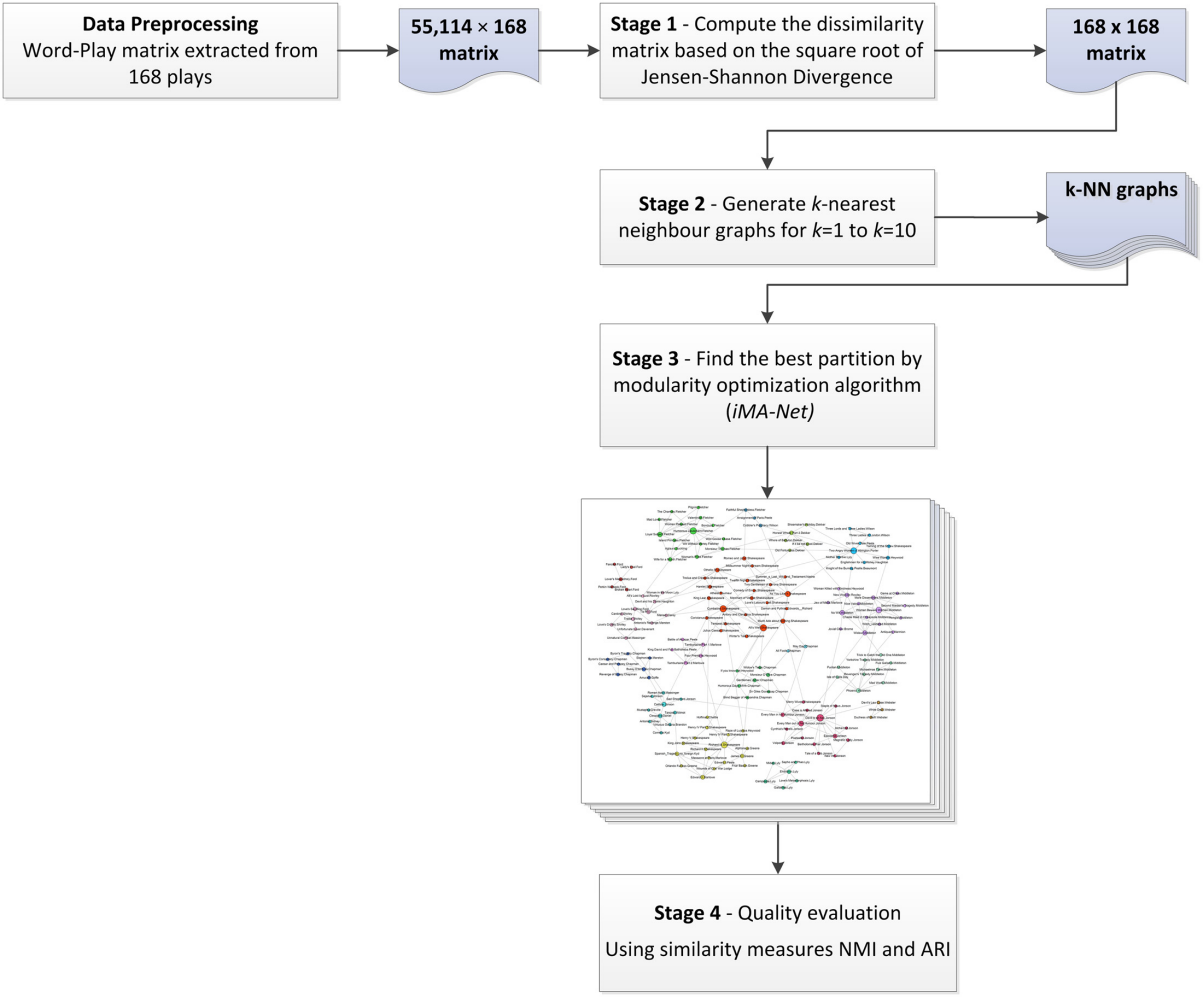
Flowchart of stages in the clustering methodology proposed in this study.

As mentioned earlier, to detect clusters of plays that are more similar in the given dataset, we propose an unsupervised graph-based clustering methodology that employs a new memetic algorithm which optimizes the modularity value in *k*-Nearest Neighbour (kNN) graphs derived from the dataset. Fig 1 outlines our methodology as a four-stage process. This clustering methodology is general; it can be used to detect clusters in a dataset of *n* elements where each element has *m* quantified features (the input dataset can be represented in a form of a (*m* × *n*) matrix). In the current study, *n* equals to 168 and refers to the number of plays and *m* equals 55,114 and refers to the words used in the plays. Details of each stage are described in the following section. The primary idea of this partitioning approach was proposed in [33] as a new method to analyse a complete network derived from field survey aimed at detecting consumer communities of trust and confidence in the Australian not-for-profit and charity sector. In this paper, we extended the primary idea by using the square root of Jensen-Shannon divergence metric ($\sqrt{JSD}$) as the dissimilarity metric, enhancing the modularity optimization algorithm and finding clusters with higher quality.

#### Stage 1- Compute the Dissimilarity Matrix

We start our method by computing the dissimilarity between all pairs of plays. We use the square root of Jensen-Shannon divergence ($\sqrt{JSD}$). JSD is a metric measuring the dissimilarity between two probability distributions based on Jensen’s inequality [34] and the Shannon entropy [35]. We refer to Lin [36] for a discussion of the JSD. The JSD has been used in many studies, especially in bioinformatics [37–39]. The $\sqrt{JSD}$ is selected being the dissimilarity metric because it has the distance metric properties and satisfies the *triangle inequality* condition (this property is proved in [40, 41]). The JSD reflects the dissimilarity of two plays based on the frequency of different words appeared in each play. Generally, for two probability distributions, *P* and *Q*, JSD is computed as follows,

$$JSD\left(P,Q\right)=H\left(\frac{P+Q}{2}\right)-\frac{H(P)+H(Q)}{2}$$ (1)

where, *H*(*X*) is Shannon information entropy for distribution *X* and is computed by Eq 2.

$$H\left(X\right)=-\sum_{x_{i}\in X}x_{i}\log_{2}x_{i}.$$ (2)

Here, *x*_*i*_ is the probability of occurrence of word *i* in the play *X*. The JSD is a non-negative and symmetric metric, therefore, *JSD*(*P*, *Q*) = *JSD*(*Q*, *P*). For two identical probability distributions (i.e. *P* = *Q*), *JSD*(*P*, *Q*) = 0, i.e. there is no dissimilarity between the two. Moreover, by using the *log*_2_ in Eq 2 the *JSD* is bounded by 1, thus $0\leq \sqrt{JSD}\leq 1$.

By computing the $\sqrt{JSD}$ for all pairs of plays, we obtain a symmetric 168 × 168 matrix representing the dissimilarity between all plays (see S1 Table). In this study, the $\sqrt{JSD}$ matrix is computed using the cibm.utils package developed in R. A graph is associated with the dissimilarity matrix, where each node represents a play and edges connect nodes where the weight of an edge is the dissimilarity between the plays. In the next stage, we will introduce a new method to analyze this graph.

#### Stage 2- Generate *k*-Nearest Neighbour (kNN) Graphs

In this stage we generate incomplete graphs by removing edges that are less important from the complete graph and preserving edges that represent stronger connections between nodes. For this purpose, we set up a series of kNN graphs starting with *k* = 1 to *k* = 10 (in total 10 graphs). To generate the kNN graphs from the dissimilarity matrix, a pair of nodes (*a*, *b*) is connected if either *a* is one of the *k*-nearest neighbors of *b* or if *b* is among the *k* nearest neighbors of *a*. The kNN graphs of this study are generated by the scalable software tool proposed by Arefin et al. [42].

Investigating 10 different kNN graphs gives us the opportunity to study the performance of our method under different cases. By increasing the value of *k*, the average degree of nodes is increased and the graph becomes denser. Therefore, identifying meaningful clusters in kNN graphs with a larger *k* is more computationally challenging in practice, as graph clustering methods identify clusters in the graph based on the graph’s edge structure, and more edges simply means more possible solutions that the method has to analyze. Table 2 shows the number of edges and the average degree for each of the kNN graphs.

**Table 2:** Basic information of kNN graphs constructed from the complete dataset. All graphs have 168 nodes that are representing the 168 plays in the dataset.

kNN Graphs	# Edges	Average Degree
k = 1	145	1.7
k = 2	284	3.4
k = 3	441	5.3
k = 4	551	6.6
k = 5	687	8.2
k = 6	822	9.8
k = 7	957	11.4
k = 8	1,091	13.0
k = 9	1,225	14.6
k = 10	1,362	16.2

We then apply a clustering approach to the kNN graphs. To detect the clusters, we developed a memetic algorithm that optimizes modularity value as the evaluation criterion. Due to the fact that the memetic algorithm uses the edge weights to cluster nodes with strong connections, we have to modify the edge weights in kNN graphs in order to represent the ‘similarity’ between nodes so that the clusters will contain similar nodes. For this purpose, similarity is defined as one minus dissimilarity.

#### Stage 3- Modularity optimization algorithm

Modularity optimization is one of the most popular methods for detecting graph structure and hidden groups of nodes with many intra-connections and comparatively few inter-connections. As explained in the introduction, such a group of nodes in the graph is called a community or cluster. It is believed that nodes which belong to the same community (cluster) are more likely to play a similar role or share common functionality [18, 19]. Detecting the community structure of a given graph, or *community detection*, is a newer nomenclature for the *graph clustering problem* and both problems are similarly looking for groups of “related” nodes to label as a cluster or community [14]. It is worth emphasizing that detecting the community structure is practicable only if graphs are *sparse* [19]. If the number of edges is close to the maximal number of edges, then the graph is a *dense* graph, the nodes become too homogeneous and the community structure does not make sense turning the problem into *data clustering*, which has its own concepts and methods that are more suitable for high density graphs [5, 43].

As a variety of complex systems in biology, sociology, physics and computer science can be represented as graphs, community detection is one of the most attractive problems in many disciplines [44, 45]. Although different approaches and methods have been proposed to solve the community detection problem, it is still a difficult problem and has not been solved satisfactorily [19]. Modularity, which was first introduced by Newman and Girvan [17], has the ability to evaluate the quality of communities, therefore it has become the key objective function in most of the recent algorithms.

The modularity function is formulated by computing the difference between the number of the edges within a community (i.e. sub-graph) and the number of edges expected to appear in the same sub-graph in the *‘null-model’*. The null-model is a model of the graph which is used for comparison, to verify whether the graph has community structure. The most popular null-model is the randomised version of the original graph proposed by Newman and Girvan, where the same number of nodes are randomly assigned, under the constraint that the expected degree of each node matches the degree of the node in the original graph [19, 46]. The random graph is not expected to have a community structure, therefore the graph has a community structure if it is significantly different from the random graph. In other words, higher modularity shows greater difference between the edge density in a sub-graph and expected value in the random graph, revealing community structure. Given an edge-weighted graph *G* = (*V*, *E*, *W*) the modularity *Q* of a clustering solution (i.e. community structure) *I* is defined as follows:

$$Q\left(I\right)=\sum_{c\in I}\left(\frac{l_{c}}{M}-{\left(\frac{d_{c}}{2M}\right)}^{2}\right)$$ (3)

The summation runs over all the communities *c* contained in the solution *I*, and *M* refers to the total weight of *G*, i.e.

$$M=\sum_{e_{ij}\in E}w_{ij},$$ (4)

where *w*_*ij*_ denotes the weight of the edge connecting node *i* and node *j*. In Eq 3, *l*_*c*_ stands for the total weights of edges connecting two nodes in community *c* and *d*_*c*_ is the sum of the weighted degrees of the nodes in community *c*, i.e.

$$l_{c}=\sum_{i,j\in c}w_{ij}\;$$ (5)

$$d_{c}=\sum_{i\in c}\sum_{\forall j}w_{ij}$$ (6)

A solution with a higher value of modularity is believed to indicate a better community structure or clustering solution within the given graph. Therefore, the maximization of modularity is by far the most popular approach for identifying the community structure of a graph [19]. Since Brandes et al. [47] proved that modularity maximization leads to a NP-complete problem, finding the community structure with the maximum modularity has become a great challenge for data scientists. Therefore, many heuristic algorithms have been developed to find near optimal and good solutions. Some of the most popular methods employed for modularity maximization are heuristics based on hierarchical or aggregation clustering methods [17, 48–50], genetic algorithms [51, 52], memetic algorithms [53–55], spectral methods [56, 57] and simulated annealing [58].

In the present study, we propose a new memetic algorithm specially designed for solving the community detection problems named “iMA-Net” that is an improved version of the MA-Net algorithm previously proposed in [59]. There are three main differences between MA-Net and iMA-Net. 1) MA-Net considers all graphs as unweighted graphs while iMA-Net detects communities in weighted graphs. 2) iMA-Net employs an enhanced local search algorithm to make the algorithm more stable. 3) While the population initialization procedure in both MA-Net and iMA-Net applies local search to each individual to improve its fitness, differing local searches make the initialization procedure performance different in MA-Net and iMA-Net. The framework and steps of iMA-Net are explained in detail in the following sections. In the last section of stage 3, the experimental results of the proposed algorithm (iMA-Net) in five real-world benchmark networks are given. To illustrate the performance reliability of iMA-Net, we compare results of iMA-Net with MA-Net [33] and those from five representative community detection algorithms.

#### iMA-Net: A Memetic algorithm for Modularity optimization

Due to the proven success of memetic algorithms and their effectiveness in dealing with many NP-hard combinatorial optimization problems [60], we chose the memetic framework for our modularity optimization algorithm, named iMA-Net. iMA-Net is a population-based algorithm in which each solution in the population represents a particular clustering of the given weighted and undirected graph. In the proposed algorithm, solutions are evolved using problem-specific genetic operators and a local search procedure employed to detect high-quality community structure with the optimal or near optimal modularity value.

**Fig 2:**
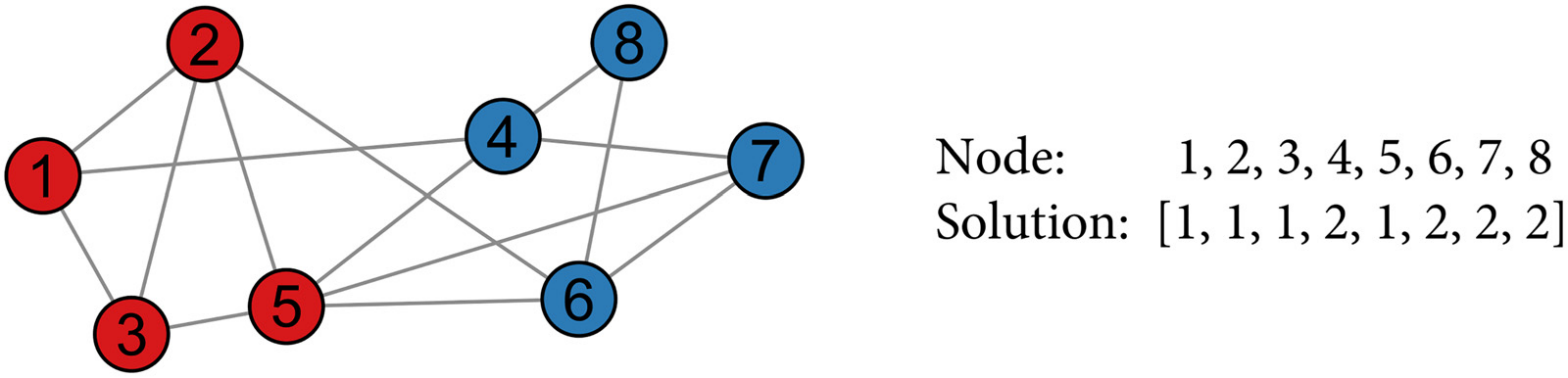
An illustration of the string-coding representation for a clustering solution. Left: a simple toy graph with two clusters shown using different colors, Right: an integer list encoding the solution, where the red and blue color clusters are labeled as 1 and 2, respectively.

We used the *string-coding* solution representation in which a clustering solution of *G* is represented by a list of *n* integer numbers [*C*_1_, *C*_2_, …, *C*_*n*_], where *n* is equal to the number of nodes in *G* and *C*_*i*_ refers to the community label of node *v*_*i*_. Therefore, if *C*_*i*_ = *C*_*j*_ then *i* and *j* are located in the same community. This way of representing clustering solutions has been used in previous algorithms, for instance [51, 53, 54]. An illustration example of string-coding representation is given in Fig 2.

Algorithm 1 presents the overall framework of iMA-Net for finding the partition of *G* that achieves the maximum modularity (computed following Eq 3). Details of each step of the algorithm are explained in the following paragraphs.

**Algorithm 1. iMA-Net Framework**

**Input:** Graph *G* = (*V*, *E*, *W*)

**Parameters:** *N*_*p*_: Population size, $P_{m}^{0}$: Minimum mutation probability, *N*_*s*_: Acceptable number of generations without improvement.

**Output:** I* a partition of *G* with fitness value Q*

*pop* = {*I*_1_, *I*_2_, …, $I_{N_{p}}$} ← Initialization(*G*,*N*_*p*_);

**repeat**

 *I*_*new*_ ← Modularity Based Recombination(*pop*);

 *I*_*mut*_ ← Adaptive Mutation(*I*_*new*_, $P_{m}^{0}$, *N*_*s*_);

 *I*_*imp*_ ← Local Search(*I*_*mut*_);

 *pop* ← Update(*pop*, *I*_*imp*_);

**until** *Termination Condition*(*N*_*s*_);

**return** *I* * = *argmax*_*I* ∈ *pop*_{*Q*(*I*)};

**Initialization:** The initial population consists of *N*_*p*_ feasible solutions, so the following process runs *N*_*p*_ times to build the initial population. Firstly, each of the *n* nodes in *G* is given a random community label that can be any integer number in the range [1,*n*]. Then to improve the quality of the solutions in the initial population, the local search procedure, as explained later in this section, is applied on each solution and made changes on community labels of nodes to find a neighbor solution with higher modularity. As the community label is selected randomly from a wide range of [1,*n*], there is not any constraint on the number of the different labels or the number of clusters in a solution. Applying the local search procedure on each solution, improves the quality of the initial population and speeds up the convergence of the algorithm.

**Modularity-based recombination operator:** Traditional operators including uniform, one-point and two-point recombination operators are not suitable for the string based representation as they do not convey characteristics of parent solutions to the offspring. iMA-Net uses a problem-specific recombination operator for generating new solutions that inherit the best characteristics from the parent solutions which is named the *modularity-based recombination operator*. This recombination operator was first proposed for MA-Net [59]. This recombination method effectively transfers the best characteristics of the parents to the descendants and it works as follows.

First, two solutions *I*_*a*_ and *I*_*b*_ are selected uniformly at random from the population as parents. The initialization procedure allows us to safely assume that all population members have relatively good quality and the random selection strategy helps maintain the population diversity. Next, a priority list *PL* is generated by sorting all communities in both parents based on their fitness values, as computed by Eq 3. Then, the best community with the highest priority from *PL* is selected and the same community is formed in the offspring. Then, for the next community in the priority list, form the most similar community in the offspring with the nodes that are not assigned to any community yet. This procedure runs until all the nodes in the offspring solution have been assigned to a community.

The offspring generated by the modularity-based recombination operator inherits the best community structure from their parents [33]. One of the limitations of modularity optimization algorithms is their difficulty in detecting small size communities and their tendency to merge small communities and form large communities [61]. The modularity-based recombination operator that we proposed has the advantage of generating offspring with a larger number of communities than its parents. This advantage helps the algorithm to explore the search space more deeply and not avoid solutions with small communities.

**Adaptive mutation operator:** This operator changes the community’s label of nodes with the probability of *P*_*m*_. This means that in each run of the adaptive mutation operator ⌈*P*_*m*_ * *n*⌉ nodes are randomly moved to one of their neighbors’ communities. Thus, this mutation operator is neighbor-based and considers only effective changes. The mutation probability (*P*_*m*_) is adaptive and it linearly grows from $P_{m}^{0}$ to $2P_{m}^{0}$, as the algorithm approaches the termination condition. As shown in Algorithm 1, the stopping condition of iMA-Net is *N*_*s*_ generations without any improvement in the best solution found so far. By increasing the mutation probability (*P*_*m*_), more changes will occur in the solution, thus diversity of the population will grow and the search area will expand. For instance, when the algorithm’s parameters are set to be $P_{m}^{0}=0.05$ and *N*_*s*_ = 30 and the algorithm runs 15 generations without improvement in the best solution found, the mutation probability will grow to $\frac{15}{30}P_{m}^{0}+P_{m}^{0}$ and it will be *P*_*m*_ = 0.075.

**Local search:** The local search procedure works on every new solution developed by the adaptive mutation operator, and it aims to improve the fitness of the solution before adding it to the population. iMA-Net uses the Vertex Movement (VM) heuristic [62] together with a stochastic hill climbing strategy to search the neighborhood to find a better solution. The local search procedure is shown in 2. Given a graph *G* with *n* nodes, the local search procedure works as follows. Firstly, *L*, a random sequence of the nodes is generated. Then, for each node *v*_*i*_ chosen according to *L*, *v*_*i*_’s label is changed to that of a randomly selected neighbor, if the change improves the fitness of the solution. If the movement of *v*_*i*_ from its community to none of its neighbors’ community increases the fitness (modularity), we leave *v*_*i*_ in its place and check the next node in *L*. The above process runs until there is no change for any of the nodes in *L* that improves the fitness of the solution (see Algorithm 2).

**Algorithm 2. Local search procedure**

**Input:** Graph *G* with *n* nodes; A clustering solution *I* = [*C*_1_, …, *C*_*n*_].

**Output:** $I^{{}^{\prime }}=\left[C_{1}^{{}^{\prime }},...,C_{n}^{{}^{\prime }}\right]$ a solution with better fitness than *I*.

*I*′ ← *I*;

**repeat**

 *t* = 0 (*t* shows the number of nodes that cannot move);

 *L* ← a random sequence of *n* nodes;

 **for** *each node* *v*_*i*_ *in* *L* **do**

  *N* ← a list of *v*_*i*_’s neighbours located in other communities;

  Δ*Q* = 0;

  **while** Δ*Q* ≤ 0 **do**

   *v*_*j*_ ← a neighbour node selected from *N*;

   Δ*Q* ← fitness change due to the movement of *v*_*i*_ to the community of *v*_*j*_;

  **end**

  **if** Δ*Q* > 0 **then**

   $C_{i}^{{}^{\prime }}=C_{j}$ (*v*_*i*_ is moved to the community of *v*_*j*_);

  **else**

   *t* = *t* + 1 (when *v*_*i*_ is not moved);

  **end**

 **end**

**until** *t* = *n*;

**return** *I*′;

The local search procedure is designed to accept the first random movement of the node that improves the fitness value. This stochastic behavior in selecting the neighbor helps the algorithm to avoid one of the known limitations of deterministic hill climbing strategies which is getting stuck in the local optima. The local search procedure operation is effective and we reduced the computational cost by using the method proposed in [50] for computing the change to the modularity Δ*Q* incurred moving node *v*_*i*_ into the community of node *v*_*j*_.

**Elitist population updating strategy:** As with MA-Net [33], in iMA-Net the population updating strategy is elitist. New solutions generated by the local search procedure are added to the population and the least fit solutions are removed from the population. The elitist strategy enhances the algorithm convergence by retaining the better solutions in the population, making them eligible to be parents in the subsequent generations.

#### Performance results of iMA-Net on benchmark networks

Before applying the iMA-Net on the kNN graphs produced in Stage 2, we tested the performance of iMA-Net on five real-world networks with similar size to the kNN graphs. These networks include the Zachary’s Karate Club network (karate) [63], the Bottlenose Dolphins network (dolphin) [64], the American College Football network (football) [18], the Political Books network (polbooks) [56] and the Jazz Musicians network (jazz) [65]. Basic information on these benchmark networks is shown in Table 3. All of these networks are unweighted and undirected graphs.

**Table 3:** Basic information of the real-world networks.

	Network	# Nodes	# Edges	Average Degree
1	karate	34	78	4.59
2	dolphin	62	159	5.13
3	polbooks	105	441	8.40
4	football	115	613	10.66
5	jazz	198	2742	27.70

The comparison between iMA-Net and MA-Net in Table 4, is made to illustrate the effectiveness of iMA-Net. Moreover, to investigate the performance of iMA-Net, we compared the iMA-Net results with a set of well-known community detection algorithms with different approaches, frameworks and objective functions. The benchmark algorithms include LPAm [66], Meme-Net [67], MOGA-Net [68], MODPSO [69] and MLCD [70].

**Table 4:** Experimental results on five real-world benchmark networks. The maximum, average and standard deviation of modularity values (*Q*_*max*_,*Q*_*avg*_,*Q*_*std*_) obtained by LPAm, Meme-Net, Moga-Net, MODPSO, MLCD, MA-Net and iMA-Net.

	Criterion	LPAm	Meme-Net	Moga-Net	MODPSO	MLCD	MA-Net	iMA-Net
karate	*Q*_*max*_	0.4052	0.4020	0.4159	**0.4198**	**0.4198**	**0.4198**	**0.4198**
	*Q*_*avg*_	0.3564	0.4020	0.3945	0.4182	**0.4198**	0.4195	**0.4198**
	*Q*_*std*_	0.0285	0	0.0089	0.0079	0	0.0022	0
dolphin	*Q*_*max*_	0.5071	0.5185	0.5034	0.5264	**0.5285**	**0.5285**	**0.5285**
	*Q*_*avg*_	0.4938	0.5096	0.4584	0.5255	**0.5285**	0.5247	0.5252
	*Q*_*std*_	0.0114	0.0061	0.0163	0.0070	0	0.0032	0.0026
polbooks	*Q*_*max*_	0.5145	0.5232	0.4993	0.5264	**0.5272**	**0.5272**	**0.5272**
	*Q*_*avg*_	0.4976	0.5218	0.4618	0.5263	**0.5272**	0.5255	0.5270
	*Q*_*std*_	0.0158	0.0031	0.0129	0.0007	0	0.0029	0.0004
football	*Q*_*max*_	0.6032	0.6044	0.4325	**0.6046**	**0.6046**	**0.6046**	**0.6046**
	*Q*_*avg*_	0.5777	0.6023	0.3906	0.6038	**0.6046**	0.5984	0.6042
	*Q*_*std*_	0.0199	0.0015	0.0179	0.0011	0.0000	0.0051	0.0006
jazz	*Q*_*max*_	0.4448	0.4376	0.2929	0.4421	**0.4451**	**0.4451**	**0.4451**
	*Q*_*avg*_	0.4360	0.4330	0.2952	0.4419	0.**4451**	0.4448	0.4450
	*Q*_*std*_	0.0098	0.0011	0.0084	0.0001	0.0000	0.0002	0.0001

LPAm presented by Barber and Clark [66] is a label propagation algorithm which reformulated the original label propagation algorithm (LPA) [71] to overcome its drawbacks. LPAm employs a modularity-specific rule for label propagation and updates the community label of nodes repeatedly till no possible improvement can be found. Meme-Net [67] is a memetic algorithm optimizing the modularity density to discover communities in the graph. MOGA-Net [68] and MODPSO [69] are two multi-objective optimization algorithms for community detection. MOGA-Net maximizes the intra-community links and minimizes the inter-community links, while MODPSO maximizes the internal link density and minimizes the external link density. MLCD is a novel memetic algorithm presented by Ma et. al [70]. MLCD has a multi-level learning framework for detecting the community structure by optimizing the modularity with the aid of a special hybrid global-local heuristic search procedure.

In a comprehensive study [70], Ma et. al. ran all of the selected benchmark algorithms 50 times each, with the same key parameters, on several real-world and computer synthesized benchmark networks, and recorded the maximum, average and standard deviation of the modularity values (*Q*_*max*_,*Q*_*avg*_,*Q*_*std*_). In this study, we refer to the reported results in [70] and compare results obtained by 50 independent runs of MA-Net and iMA-Net with the benchmark algorithms. MA-Net and iMA-Net are implemented in Python 2.7 and executed on a PC with Intel ^®^ Xeon ^®^ CPU E5-1620 at a clock speed of 3.6 GHz (4 cores and 8 logical processors) and 16 GB of memory. We tuned the parameters of MA-Net and iMA-Net as follows: population size, *N*_*p*_, is set to 40, the minimum mutation probability, $P_{m}^{0}$, is set to 0.05 and the termination condition is set to 30 generations without improvement (*N*_*s*_ = 30). The comparison results are shown in Table 4.

Firstly, the comparison results in Table 4 show that in all benchmark networks MLCD, MA-Net and iMA-Net obtained the largest *Q*_*max*_. While both MA-Net and iMA-Net achieved the maximum possible modularity in all networks, iMA-Net has more reliable performance because it improves the *Q*_*avg*_. Meanwhile, comparing *Q*_*std*_ values obtained by MA-Net and iMA-Net show that in all networks iMA-Net has smaller deviation, indicating that the improvements in iMA-Net enhance the stability and accuracy of the algorithm. Comparisons between the *Q*_*avg*_ values obtained by iMA-Net and the other algorithms show that in all cases iMA-Net achieved higher *Q*_*avg*_ and performed better than four representative algorithms, LPAm, Meme-Net, Moga-Net and MODPSO. Only MLCD, which is a novel multi-level learning memetic algorithm has a better performance than iMA-Net in benchmark networks. As the MLCD method is not publicly available and iMA-Net shows outstanding performance in detecting the high quality community structure with great stability, in this study we use the iMA-Net as the community detection algorithm. It is important to note that the clustering methodology is a generic method and any community detection algorithm can be employed in the third stage.

#### Stage 4- Partition Quality Evaluation

To evaluate the quality of the solution obtained by our clustering method, we can compare the solution with the known authorship label of each play. In other words, we expect that a good solution would put the plays written by the same author in one group. Thus, we are considering the authorship as the true label of each play and to evaluate the solution quality we used two well-known clustering similarity measures: *Normalized Mutual Information* (NMI) and *Adjusted Rand Index* (ARI).

**NMI:** A symmetric index computing the similarity between two clustering solutions based on the confusion matrix (also referred to as the contingency matrix). As defined in [72], for two clustering solutions of a given graph *A* and *B*, the NMI is defined as follows:

$$NMI\left(A,B\right)=\frac{-2\sum_{i=1}^{c_{A}}\sum_{j=1}^{c_{B}}F_{ij}\log\left(\frac{F_{ij}.n}{F_{i}^{row}\cdot F_{j}^{col}}\right)}{\sum_{i=1}^{c_{A}}F_{i}^{row}\log\left(\frac{F_{i}^{row}}{n}\right)+\sum_{j=1}^{c_{B}}F_{j}^{col}\log\left(\frac{F_{j}^{col}}{n}\right)}$$ (7)

where *F* is the confusion matrix for solutions *A* and *B* in which rows and columns correspond to the clusters in *A* and *B*, respectively. *F*_*ij*_ is the node-overlap *i*th cluster of *A* and *j*th cluster of *B*. $F_{i}^{row}$ is the sum of the elements in the row *i* of *F* and $F_{j}^{col}$ denotes the sum of elements in the column *j* of *F*. *c*_*A*_ and *c*_*B*_ are the number of clusters in *A* and *B*, respectively. The NMI measures the similarity of two solutions, therefore if *A* = *B*, then *NMI*(*A*,*B*) = 1 and if there is no similarity between *A* and *B* then *NMI*(*A*,*B*) = 0.

**ARI:** The adjusted version of the *Rand Index*(RI), first introduced in [73], compares two clusterings based on the number of cluster membership agreements and disarrangements between them. It shows the ratio of the number of node pairs similarly classified in both solutions, divided by the total number of pairs. RI is defined as

RI(A,B)=α+βn2, (8)

where *n* is the number of nodes, *α* refers to the number of pairs of nodes which are in the same cluster in solution *A* and in the same cluster in solution *B* and *β* is the number of pairs of nodes that are classified in different clusters in both solutions. The RI lies between 0 and 1 and it is equal to 1 when two solutions are exactly the same. The ARI was proposed by Hubert and Arabie [74]. To adjust the RI they assumed the generalized hypergeometric distribution as a null model and defined ARI as follows:

ARI(A,B)=∑i,jFij2-∑iFirow2∑jFjcol2/n212∑iFirow2+∑jFjcol2-∑iFirow2∑jFjcol2/n2 (9)

where, similar to Eq 7, *F*_*ij*_, $F_{i}^{row}$ and $F_{j}^{col}$ are extracted from the confusion matrix *F*. Like the NMI, a larger ARI shows that two solutions are more similar. The ARI attracted more attention than RI as it is in the range [−1, +1], the wider range of values increasing the sensitivity of the index. To combine the effectiveness of NMI and ARI, we also used their product, NMI × ARI, for evaluation and comparing different clusterings.

## Results

We applied iMA-Net to a series of ten kNN graphs (for *k* = 1 to *k* = 10) to optimize the modularity value and find the best solution that partitions the set of 168 plays written by 39 authors. The computer used for these experiments has the same configuration as stated in the section ‘Performance results of iMA-Net on real-world networks’. We ran iMA-Net 30 times for each graph with the following parameters: population size *N*_*p*_ is set to 40, the termination criterion is set to 30 generations without improvement (*N*_*s*_ = 30) and the minimum mutation probability is equal to 0.05 ($P_{m}^{0}$). The best solution found in 30 runs of the algorithm, which has the highest value of modularity, is recorded in Table 5. Quality measures including NMI, ARI and NMI × ARI, representing the similarity between best solutions and the true clustering solution obtained from the authorship labels, are shown in Table 5.

**Table 5:** The best solution found by iMA-Net in ten kNN graphs derived from 168 Shakespearean era play dataset.

kNN graph	Q	# Clusters	NMI	ARI	NMI×ARI
**k = 1**	0.898	24	0.686	0.317	0.217
**k = 2**	0.751	18	**0.742**	**0.525**	**0.390**
**k = 3**	0.713	10	0.690	0.457	0.315
**k = 4**	0.670	9	0.673	0.441	0.296
**k = 5**	0.623	9	0.663	0.431	0.286
**k = 6**	0.579	9	0.670	0.429	0.288
**k = 7**	0.542	9	0.672	0.434	0.292
**k = 8**	0.509	8	0.639	0.396	0.253
**k = 9**	0.482	8	0.639	0.401	0.256
**k = 10**	0.455	8	0.623	0.392	0.244

**Q** is the modularity value used as a fitness value of the clustering. **NMI, ARI** and **NMI×ARI** are quality measures used to compare with the true solution of the dataset based on the authorship of plays.

**Fig 3:**
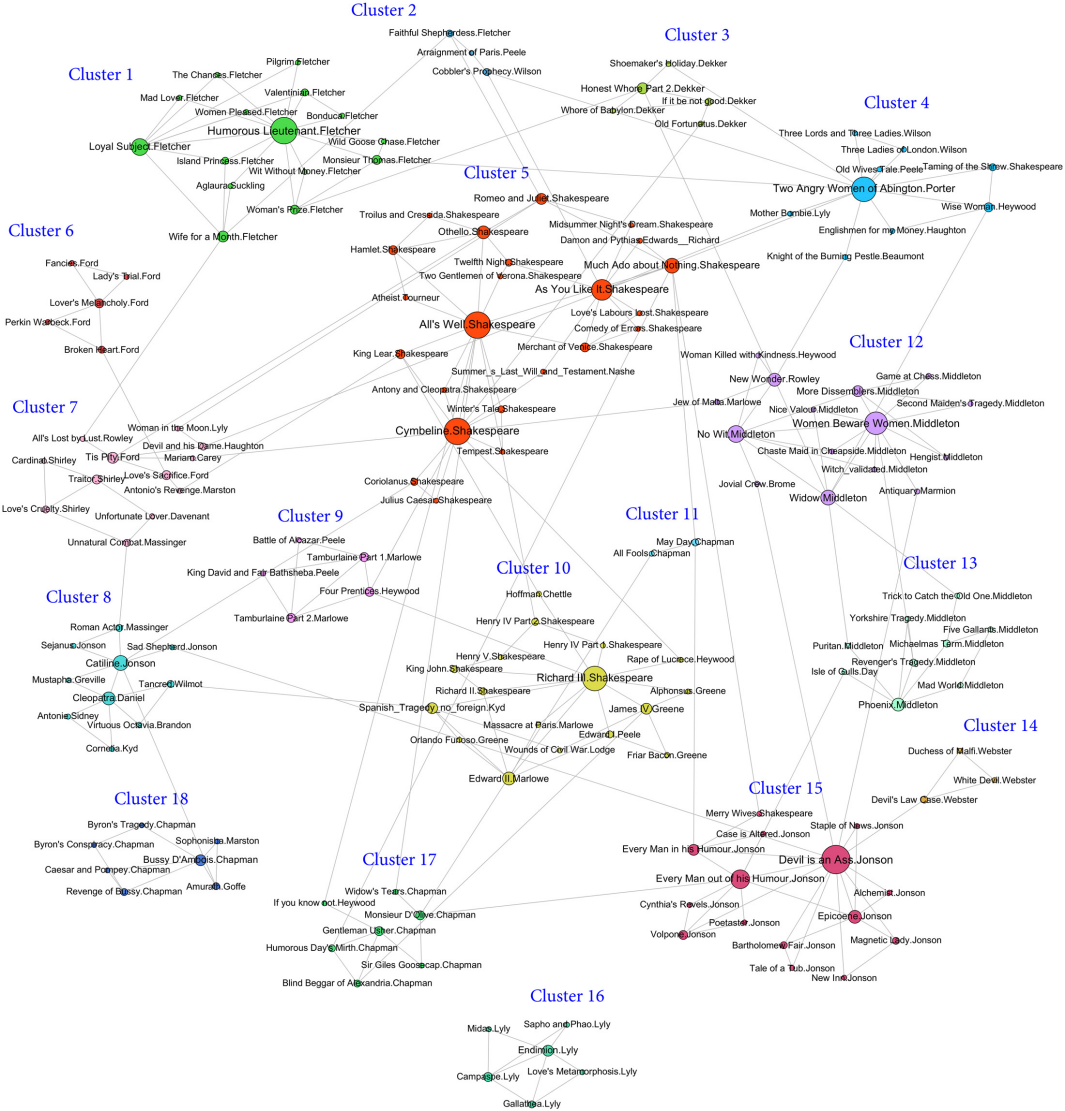
Best clustering outcome of iMA-Net with the highest NMI and ARI. 18 clusters are detected in 2-nearest neighbor graph shown by different colours. Node size is proportional to the node’s total degree. Nodes are labeled by the play.author.

The results in Table 5 demonstrate that by increasing the value of *k* and adding more connections to the graph, the number of the detected clusters and the solution modularity (*Q*) is reduced. Because of the dependency of the highest value of *Q* on the structure of the graph, it cannot be used for comparing solutions from different kNN graphs. But we can refer to quality measures (NMI and ARI) to find in which graphs iMA-Net finds the solution that better matches authorship labels. The results show that the best-matched solution with the highest quality measures, i.e. NMI = 0.742 and ARI = 0.525, is obtained by analyzing the 2-nearest neighbor (2NN) graph and contains 18 clusters, as is shown in Fig 3.

Fig 3 presents the best solution in 2NN graph where each node represents a unique play. The play’s author is shown as the label of the node and the detected clusters are identified by different colors. Lists of the plays located in each cluster of this clustering solution together with the confusion matrix of the solution and the true solution is available in S2 and S3 Tables.

Moreover, Fig 3 shows that the solution contains five clusters dedicated to only one author, where all plays in these clusters are written by the same author. The five finely detected authors are Dekker (Cluster 3), Ford (Cluster 6), Lyly (Cluster 16), Chapman (Cluster 11) and Webster (Cluster 14). It also contains a good classification of authors with more contributions in the dataset; for instance the biggest cluster (Cluster 5) with 23 plays includes 20 plays attributed to Shakespeare together with three plays from authors that have only one contribution in the dataset. Moreover, we have a very good clustering of the plays written by four authors Fletcher (Cluster 1), Middleton (Cluster 13), Jonson (Cluster 15) and Chapman (Cluster 17). These authors are separately clustered excepting only one play by another author included in each cluster.

Furthermore, considering authors with limited contributions in this dataset and the way these works grouped with other plays reveals some interesting insights about their literary style. For instance, the single Tourneur play in the dataset is classified in Cluster 5 with 20 plays of Shakespeare. Commentators have seen Tourneur as a follower of Shakespeare [75], and it is likely that in this case the influence of Shakespeare’s style explains the placement of the play within the cluster. Another example is the clustering of the play of Marmion and the single play of Brome in the same group (Cluster 12) with 10 plays attributed to Middleton that illustrate the similarity between their word usage.

### Reduced Datasets

Referring to Table 1, the complete dataset contains 19 authors with only one attributed play. iMA-Net classified these plays with the most similar plays, maximizing the modularity value. However, this behavior reduces the quality measures (NMI and ARI). To better understand the performance of the proposed methodology, we applied the same stages on reduced datasets resulting from removing some plays from the original dataset. The five *reduced datasets* generated by removing plays written by authors who have 1, 2, 3, 4 and 5 contributions are as follows:

1. **G1**: Plays from authors with more than one contribution.
2. **G2**: Plays from authors with more than two contributions.
3. **G3**: Plays from authors with more than three contributions.
4. **G4**: Plays from authors with more than four contributions.
5. **G5**: Plays from authors with more than five contributions.

Table 6 shows the number of plays and authors that remained in each of the reduced datasets. We applied our four-stage method to the five reduced datasets (**G1-G5**) and the results are given in Table 7.

**Table 6:** Configuration of reduced datasets.

Reduced dataset	# Plays	# Authors
**G1**	149	20
**G2**	139	15
**G3**	130	12
**G4**	126	11
**G5**	106	7

**Table 7:** Best solutions found by iMA-Net in 10 kNN graphs derived from each reduced dataset (G1-G5). The highest values of NMI, ARI and NMI×ARI in each dataset are denoted in bold.

Reduced dataset	kNN graph	Q	# Clusters	NMI	ARI	NMI×ARI
**G1**	**k = 1**	0.907	23	0.696	0.371	0.258
	**k = 2**	0.753	16	**0.738**	0.505	0.373
	**k = 3**	0.713	10	0.659	0.451	0.297
	**k = 4**	0.672	9	0.701	0.505	0.354
	**k = 5**	0.625	10	0.711	**0.542**	**0.386**
	**k = 6**	0.583	9	0.669	0.457	0.306
	**k = 7**	0.541	9	0.676	0.463	0.313
	**k = 8**	0.511	8	0.628	0.415	0.261
	**k = 9**	0.476	7	0.642	0.438	0.281
	**k = 10**	0.447	8	0.629	0.424	0.267
**G2**	**k = 1**	0.903	22	0.707	0.393	0.278
	**k = 2**	0.765	16	**0.741**	0.499	0.370
	**k = 3**	0.722	11	0.717	0.522	0.374
	**k = 4**	0.680	9	0.702	0.520	0.365
	**k = 5**	0.630	10	0.717	**0.564**	**0.404**
	**k = 6**	0.588	9	0.683	0.495	0.338
	**k = 7**	0.552	9	0.672	0.492	0.331
	**k = 8**	0.506	8	0.649	0.454	0.295
	**k = 9**	0.476	8	0.683	0.501	0.342
	**k = 10**	0.453	8	0.672	0.464	0.312
**G3**	**k = 1**	0.892	18	0.660	0.377	0.249
	**k = 2**	0.767	15	0.773	0.542	0.419
	**k = 3**	0.729	10	0.711	0.552	0.392
	**k = 4**	0.682	11	0.740	0.560	0.414
	**k = 5**	0.636	10	**0.744**	**0.617**	**0.459**
	**k = 6**	0.599	9	0.694	0.542	0.376
	**k = 7**	0.561	9	0.687	0.526	0.361
	**k = 8**	0.520	9	0.678	0.509	0.345
	**k = 9**	0.488	8	0.690	0.536	0.370
	**K = 10**	0.460	7	0.632	0.448	0.283
**G4**	**k = 1**	0.893	19	0.646	0.343	0.222
	**k = 2**	0.766	15	**0.777**	0.554	0.431
	**k = 3**	0.762	16	0.772	0.552	0.426
	**k = 4**	0.681	11	0.731	0.554	0.405
	**k = 5**	0.639	10	0.740	**0.628**	**0.465**
	**k = 6**	0.604	9	0.708	0.595	0.421
	**k = 7**	0.563	9	0.689	0.536	0.369
	**k = 8**	0.523	9	0.680	0.522	0.355
	**k = 9**	0.490	7	0.631	0.431	0.272
	**k = 10**	0.464	8	0.663	0.498	0.330
**G5**	**k = 1**	0.895	17	0.733	0.451	0.331
	**k = 2**	0.781	11	0.826	0.618	0.511
	**k = 3**	0.751	9	0.758	0.587	0.445
	**k = 4**	0.701	8	0.843	0.730	0.615
	**k = 5**	0.656	7	**0.856**	0.810	**0.693**
	**k = 6**	0.627	7	0.847	**0.814**	0.690
	**k = 7**	0.584	7	0.847	**0.814**	0.690
	**k = 8**	0.544	7	0.837	0.787	0.659
	**k = 9**	0.514	6	0.799	0.690	0.551
	**k = 10**	0.482	6	0.808	0.706	0.570

From the higher values of NMI and ARI in Table 7, compared to their values in the original dataset, Table 5, we can conclude that by removing authors with fewer contributions from the original dataset, the proposed clustering method found solutions with higher quality with clusters better matched to the authors’ labels. More precisely, while the average NMI and average ARI in the original dataset are 0.670 and 0.422, respectively. The average NMI increased to 0.718 and the average value of ARI to 0.538 in the reduced datasets.

**Fig 4:**
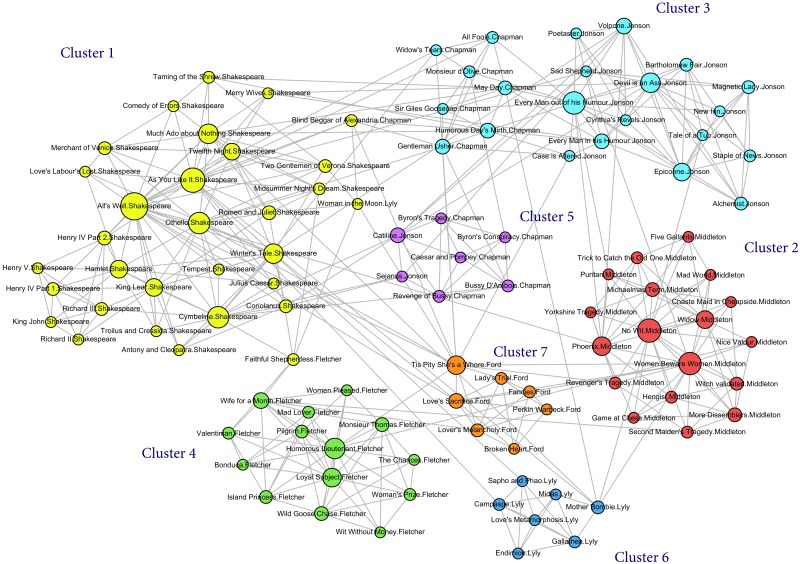
The best clustering outcome of iMA-Net with the highest NMI × ARI in G5. Seven clusters are shown by different colours in the 5-nearest neighbour graph constructed from similarity between 106 plays from 7 authors. Nodes sizes are proportioned to their degree.

On the other hand, the results in Table 7 show that NMI and ARI are different quality measures and they do not always agree on the best solution among the ten solutions found in the kNN graphs. Thus, we consider NMI × ARI to combine the effect of two measure into a single index. Interestingly, NMI×ARI always attains the highest value in the 5-nearest neighbour (5NN) graphs in all of the five reduced datasets. The best solution according to NMI×ARI is found in the **G5** dataset and in the 5NN graph where the highest NMI×ARI is equal to 0.693. Table 8 shows the confusion matrix of the true solution and the solution with the highest quality measures for NMI and NMI×ARI and Fig 4 represents this best clustering solution in the 5NN graph. For details of plays in each cluster refer to the S4 Table.

**Table 8:** Confusion matrix of the true authorship and the clustering solution obtained by the 5NN graph with *NMI* × *ARI* = 0.693. The confusion matrix shows how 106 plays by 7 authors are distributed into 7 clusters. As expected, a good separation occurred in clusters 2, 4, 6 and 7, which are formed by plays of one specific author.

	Author	# Plays	Cluster 1	Cluster 2	Cluster 3	Cluster 4	Cluster 5	Cluster 6	Cluster 7
**1**	**Shakespeare**	28	28	0	0	0	0	0	0
**2**	**Middleton**	18	0	18	0	0	0	0	0
**3**	**Jonson**	17	0	0	15	0	2	0	0
**4**	**Fletcher**	15	1	0	0	14	0	0	0
**5**	**Chapman**	13	1	0	7	0	5	0	0
**6**	**Lyly**	8	1	0	0	0	0	7	0
**7**	**Ford**	7	0	0	0	0	0	0	7
	**Total**	**106**	**31**	**18**	**22**	**14**	**7**	**7**	**7**

Both Table 8 and Fig 4 show that plays written by Middleton (Cluster 2), Fletcher (Cluster 4), Lyly (Cluster 6) and Ford (Cluster 7) are classified in completely separated clusters. All 28 plays of Shakespeare in this dataset are in the biggest group, Cluster 1, together with three plays from different authors which are the comedy *‘Blind Beggar of Alexandria’* written by Chapman, the pastoral play *‘Faithful Shepherdess’* by Fletcher and a comedy from Lyly named *‘Woman in the Moon’*. So far as we are aware no commentators have suggested previously that these three non-Shakespeare plays in Cluster 1 are particularly Shakespearean, but they are sometimes noted as unusual for their authors. In the present analysis *‘The Woman in the Moon’* is among the five nearest neighbors of *‘The Faithful Shepherdess’* and *‘The Blind Beggar of Alexandria’*, thus these three plays are themselves connected in **G5**.

The *‘Woman in the Moon’* is a departure for Lyly in that he moves to verse after a career writing prose comedies. It is his last-performed play. Critics note other important differences from Lyly’s normal practice as well [76]. None of the other seven Lyly plays in this dataset appear among the five plays with the highest similarity score and connected to *‘Woman in the Moon’*. Moreover, though this play is normally classified as a comedy [77] the closest play according to $\sqrt{JSD}$ measure is a Shakespeare tragedy, *‘Romeo and Juliet’*, and other closest is another tragedy, *‘Tis Pity She’s a Whore’*, by John Ford, from a much later phase of drama—the first production of Ford’s play was in 1632, compared to 1593 for the Lyly play. In her edition of the play Leah Scragg suggests that the play has more similarities to the rest of the Lyly canon than critics generally acknowledge, and has links to two other Lyly plays, *‘Endymion’* and *‘Mother Bombie’*, in particular [76]; the results of the present analysis come down on the side of change rather than continuity in this play. In the context of a clustering in which author and genre likenesses come through strongly, and consistently, overall, the cross-author and cross-genre links for this play are worthy of further investigation, though that is beyond the scope of the present paper.

*‘The Faithful Shepherdess’* is a pastoral, quite unlike Fletcher’s usual work in comedy and tragicomedy. In his studies of the shares of Fletcher and Beaumont in their collaborative plays Cyrus Hoy excludes *‘The Faithful Shepherdess’* from Fletcher baselines for that reason [78]. Jonathan Hope notes Fletcher’s departure from his usual style in *‘The Faithful Shepherdess’*, which he calls “a consciously literary and deliberately archaic piece” [79]. Thus it is not surprising to find it clustered away from the main Fletcher cluster. Two Fletcher plays appear in the nearest neighbors of *‘The Faithful Shepherdess’*, suggesting that the analysis has uncovered some authorial characteristics in the play, even if they are not sufficient to have it join the Fletcher cluster (Cluster 4).

Something similar may be said about the *‘Blind Beggar of Alexandria’*, which appears with two other Chapman comedies in the list of plays with the highest similarity score. The *‘Blind Beggar of Alexandria’* is Chapman’s first performed play and has often been viewed negatively as overly farcical with an underdeveloped romantic plot [80].

It is most likely that these three plays are clustered with Shakespeare because they have weak links to their authorial canons, and the Shakespeare cluster is large and therefore likely to be diverse, with many options for links.

Two other clusters (Cluster 3 and Cluster 5) are formed by mixing all 17 of Jonson’s plays and 12 plays written by Chapman. A deeper look at the plays in these two clusters shows that the genre of the plays has a great role in putting similar works in the same cluster. The larger cluster (Cluster 3) contains 21 comedies of both Jonson and Chapman together with the only pastoral play written by Jonson named (*‘Sad Shepherd’*). However, Cluster 5 contains all of six tragedy plays written by these two authors and a historical play of Chapman which is called *‘Caesar and Pompey’*. Both Jonson and Chapman would be recognized as having drastically different styles in their own comedies compared to their own tragedies, so this would fit with general critical opinion.

Taking all the clusters found by our methodology into account, authorship is generally very strong on these measures, but a few authors have either an aberrant play or a consistent internal division by genre. Chapman has both of these attributes. The clusters identified by the proposed unsupervised method show considerable associations with the authors’ writing styles. Nevertheless and in order to validate the effectiveness of our method, we applied two available clustering methods on the dataset and compared the results.

### Performance comparison with other clustering methods

The proposed method provides solutions for the clustering of plays in the dataset based on the similarity of word frequencies in each play. To compare our method with other clustering methods, we applied six well-known distance-based clustering techniques on the Complete dataset and the five reduced datasets (**G1-G5**). The selected clustering methods for comparisons are: a well-known implementation of the k-means method named *k-means++* [81], one unsupervised graph-based clustering algorithm named *MST-kNN* [32], and four popular hierarchical clustering methods: 1)Complete-linkage, 2)Average-linkage, 3)Single-linkage and 4)Ward’s algorithm.

k-means is by far the most popular clustering method used in many scientific and industrial applications [82, 83]. k-means++ developed by Arthur [81], is a novel k-means algorithm with an enhanced seeding technique for outperforming the standard k-means. Due to the popularity of k-means methods, k-means++ is selected for comparison. k-means++ is implemented in the Scikit-learn(0.16.1) clustering package [84] in Python and is publicly available. It is worth noting that k-means++, according to the original method, uses the Euclidean distance and identifies clusters by minimizing the average square distance between elements in the same clusters [81]. This method is a supervised method, in the sense that the number of clusters (K) is known in advance; therefore, we run the algorithm for K = 2 to K = 20 and report the best solution based on the quality measure *NMI*×*ARI*.

MST-kNN is an unsupervised graph clustering technique proposed by Inostroza-Ponta et. al [32]. This method works by partitioning the minimum spanning tree using the k-nearest neighbour graph with an adaptive determination of the number of clusters. The MST-kNN is chosen for comparison because for two main reasons: 1) it can use the $\sqrt{JSD}$ matrix and compute the clusters based on this distance (dissimilarity) matrix, 2) MST-kNN also uses a similar approach of using graph structure for data clustering. This method has been employed in several studies [85–87] and it has shown remarkable results in data analysis. While MST-kNN is unsupervised, it is applied directly on $\sqrt{JSD}$ matrices and the obtained clusters is evaluated regarding the true partitions.

Hierarchical clustering methods seek the hierarchical structure of the graph by recursively partitioning nodes into clusters either in top-down fashion by dividing clusters into smaller clusters (divisive hierarchical clustering) or bottom-up fashion by merging clusters into larger cluster (agglomerative hierarchical clustering). Dissimilarity measures and linkage criteria are used in order to select which cluster should be merged or divided. The linkage criterion refers to the manner in which the distance between two clusters is calculated. The most well-known linkage criteria are complete-linkage, average-linkage and single-linkage [88]. The distance between two cluster according to the complete-linkage criterion is equal to the longest distance between any pair of nodes from the two clusters [89], but based on the single-linkage criteria it is equal to the shortest distance between any pair of nodes from the two clusters [90] and in average-linkage methods the distance between two cluster is defined as the average distance of any member of one cluster to any member of the other cluster [91].

In this study, we use the stats (3.3.0) package (hclust function) provided in R. The $\sqrt{JSD}$ is employed as the dissimilarity measure and for the linkage criterion four methods have been used: Complete-linkage, Average-linkage, Single-linkage and Ward’s method [92, 93]. Ward’s method is a modified average-linkage method that works based on the squared dissimilarity. Hierarchical methods result in dendrograms, representing the nested grouping of nodes. The clustering solution of the data elements is obtained by cutting the dendrogram at the desired level. Therefore in this study to compare the clustering result of hierarchical methods with the true partitions and other methods, the obtained dendrogram was cut to get the desired number of clusters. To give the most possible chance to the dendrogram to provide the best solution the number of clusters was varied from K = 2 to K = 20.

Table 9 shows the best and worst result of the proposed clustering methods together with the best results obtained by k-means++, MST-kNN and four hierarchical clustering methods (i.e. complete-linkage, average-linkage, single-linkage and Ward’s method). The quality of the clustering results are evaluated by NMI, ARI and NMI×ARI which are computed by comparing the clusters with the true authorship label of the plays in each dataset. The Best and the Worst results are repetitions from Table 5 (for Complete dataset) and Table 7 (for reduced datasets). Methods are ranked in each dataset based on NMI × ARI that shows how successfully the method obtained the true partition.

**Table 9:** The best clustering solutions obtained by benchmark methods (k-means++, MST-kNN, Complete-linkage, Average-linkage, Single-linkage and Ward’s method) together with the best (Best) and the worst (Worst) clustering result obtained by the proposed method in Complete dataset and five reduced datasets (G1-G5). The Rank column is based on the value of NMI × ARI.

Dataset	Method	# Clusters	NMI	ARI	NMI × ARI	Rank
**Complete**	**Best**	18	0.742	0.525	0.390	2
	**Worst**	24	0.686	0.317	0.217	3
	**k-means++**	13	0.617	0.339	0.209	4
	**MST-kNN**	2	0.292	0.026	0.008	8
	**Complete-linkage**	20	0.623	0.324	0.202	5
	**Average-linkage**	20	0.440	0.052	0.023	6
	**Single-linkage**	20	0.384	0.033	0.013	7
	**Ward’s method**	20	0.772	0.568	0.438	1
**G1**	**Best**	10	0.711	0.542	0.386	2
	**Worst**	8	0.628	0.415	0.261	3
	**k-means++**	18	0.638	0.354	0.226	4
	**MST-kNN**	3	0.435	0.090	0.039	6
	**Complete-linkage**	20	0.591	0.181	0.107	5
	**Average-linkage**	20	0.434	0.065	0.028	7
	**Single-linkage**	19	0.388	0.052	0.020	8
	**Ward’s method**	19	0.775	0.570	0.442	1
**G2**	**Best**	10	0.717	0.564	0.404	1
	**Worst**	8	0.649	0.454	0.295	3
	**k-means++**	16	0.628	0.395	0.248	4
	**MST-kNN**	3	0.469	0.132	0.062	6
	**Complete-linkage**	19	0.562	0.191	0.107	5
	**Average-linkage**	20	0.467	0.098	0.046	7
	**Single-linkage**	20	0.392	0.064	0.025	8
	**Ward’s method**	18	0.765	0.507	0.387	2
**G3**	**Best**	10	0.744	0.617	0.459	1
	**Worst**	18	0.660	0.377	0.249	4
	**k-means++**	12	0.626	0.448	0.280	3
	**MST-kNN**	3	0.476	0.147	0.070	6
	**Complete-linkage**	19	0.574	0.224	0.129	5
	**Average-linkage**	20	0.491	0.125	0.061	7
	**Single-linkage**	20	0.412	0.081	0.033	8
	**Ward’s method**	14	0.740	0.535	0.396	2
**G4**	**Best**	10	0.740	0.628	0.465	2
	**Worst**	7	0.631	0.431	0.222	4
	**k-means++**	17	0.640	0.389	0.249	3
	**MST-kNN**	3	0.486	0.162	0.079	6
	**Complete-linkage**	20	0.612	0.264	0.162	5
	**Average-linkage**	18	0.400	0.080	0.032	8
	**Single-linkage**	20	0.517	0.142	0.074	7
	**Ward’s method**	13	0.752	0.619	0.465	1
**G5**	**Best**	7	0.856	0.810	0.693	1
	**Worst**	17	0.733	0.451	0.331	3
	**k-means++**	10	0.600	0.422	0.253	5
	**MST-kNN**	3	0.540	0.237	0.128	6
	**Complete-linkage**	15	0.665	0.465	0.310	4
	**Average.linkage**	10	0.449	0.124	0.056	7
	**Single.linkage**	16	0.362	0.075	0.027	8
	**Ward’s method**	9	0.830	0.797	0.661	2

Firstly, the best results of the proposed method (Best), in the three of six datasets, i.e. **G2**, **G3** and **G5** datasets, are ranked first and outperform the other methods. In these three datasets the results of Ward’s method are in the second rank. In the other three datasets, i.e. Complete, **G2** and **G4** datasets, Ward’s method is ranked first and Best is in the second place.

Secondly, comparison between the benchmark methods shows that MST-kNN has an obvious tendency to merge small clusters. Hence, it results in a solution with fewer clusters. On the other hand, the three hierarchical clustering methods, i.e. Complete-linkage, Average-linkage and Single-linkage, result in solutions with more clusters. However, among the three traditional hierarchical clustering methods the Complete-linkage method obtained better results than Average-linkage and Single-linkage in all datasets.

Finally, comparison between the worst results of the proposed method (Worst) with other benchmark methods indicates that in four of the datasets, i.e. Complete, **G1**, **G2**, **G3** and **G5**, Worst is ranked 3 after Best and Ward’s. Another striking observation about the Worst results is that in all datasets, it is better than MST-kNN, Complete-linkage, Average-linkage and Single-linkage. Furthermore, comparison between the performance of the popular method k-means++ with Worst illustrates that in four datasets, Worst is ranked better than k-means++, and only in **G3** and **G4** does k-means++ show slightly better performance than Worst.

## Discussion

### Significance and Contribution

In this paper, we proposed a new four-stage methodology for data clustering. The methodology is unsupervised and it is able to identify groups of data elements which have a meaningful similarity. Though several methods have been proposed for two problems of *data clustering* and *community detection*, in this study, we introduced a new general methodology to convert the data clustering problem to the community detection problem by borrowing fundamental concepts of information theory and graph theory. This methodology aims to identify novel clusters from data. To evaluate the performance of our methodology, we have conducted several experiments on an interesting corpus dataset generated by counting word frequencies in 168 Shakespearean plays. Clusters obtained from datasets (Complete and reduced datasets) revealed stimulating findings about author’s literary styles.

Moreover, the memetic algorithm (iMA-Net) which is proposed optimizing the modularity value shows efficient performance in discovering solutions in benchmark networks. Also, in the kNN graphs generated for the Complete dataset and reduced datasets, the iMA-Net obtained good clustering results that are highly matched with the true partitions of the datasets. Furthermore, comparison with six well-known clustering techniques (Table 9) illustrates the great ability of the developed methodology in detecting superior clustering solutions in the studied datasets. Finally, the proposed methodology is a generic method and in each of the four stages a variety of other methods can be employed. For instance, in the third stage of identifying the community structure of the kNN graphs, variety of community detection methods might be applicable.

### Limitations and future research

Although the proposed methodology obtains remarkable results in studied datasets, there are a few limitations, which are possible areas for future work. Firstly, we utilized the square root of Jensen-Shannon divergence ($\sqrt{JSD}$) to measure the dissimilarity between elements and then convert the dissimilarity (distance) in kNN graphs to the similarity by subtracting it from one; it performs competently in the studied datasets, but more studies on different datasets and comparison with other measures are required to prove that $\sqrt{JSD}$ is the most suitable measure for constructing the kNN graphs.

Secondly, there is a limitation regarding the number of kNN graphs that should be analyzed. In the second stage of the proposed methodology, the kNN graphs were generated from the dissimilarity matrix and we set the value of *k* to be in the range of 1 to 10. As mentioned before, comparison between the best found clustering in the five reduced datasets (Table 7) showed that solutions with the highest value of NMI×ARI were always obtained from the 5NN graph. However more investigations are required to justify that analyzing 5NN graphs has an advantage over the other kNN graphs. One of the future research directions would be finding a proper procedure for tuning the value of *k* in different datasets.

Note that the proposed clustering methodology is a four-stage procedure and for each stage different tools are applicable. This has its advantages and disadvantages. While the main advantage is the flexibility of the method to employ different tools, the disadvantage is its complexity in combining many different tools. Future work will aim to develop a single software tool for the proposed clustering approach that can apply all four stages in an optimized and scalable way.

Another future research direction is to employ multi-objective community detection algorithms that optimize not only modularity value but also other quality functions. As an example, we can refer to the multi-objective community detection method proposed by Shi et al. [94]. Multi-objective algorithms return a set of non-dominated solutions and give the opportunity for the user to select the most appropriate solution from a few. Furthermore, applying overlapping community detection methods in the third stage is another important future research direction, which will enhance the ability of the proposed methodology to identify overlapping clusters; the latter has several real-world applications.

To conclude, we proposed a novel four-stage methodology for clustering based on information theory and modularity optimization. To demonstrate the effectiveness of this methodology, we conducted experiments on clustering a large text corpus dataset. We discovered remarkable results which lead to the authors’ literary styles in different genres. As discussed, we envision that the proposed efficient methodology might be adopted in various fields for the purpose of investigating the clustering structure of datasets.

## Supporting Information

S1 TableThe 168×168 matrix contains the $\sqrt{JSD}$ between each pair of 168 plays.(XLSX)

S2 Table Confusion Matrix of the best solution that is demonstrated in Fig 3. (XLSX)

S3 Table List of plays located in each cluster of the solution demonstrated in Fig 3. (XLSX)

S4 Table List of plays located in each cluster of the best clustering solution of G5 which is demonstrated in Fig 4. (XLSX)
